# Persistence of low drug treatment coverage for injection drug users in large US metropolitan areas

**DOI:** 10.1186/1747-597X-5-23

**Published:** 2010-09-21

**Authors:** Barbara Tempalski, Charles M Cleland, Enrique R Pouget, Sudip Chatterjee, Samuel R Friedman

**Affiliations:** 1 National Development and Research Institutes, Inc., 71 West 23rd Street, 8th Fl, New York, NY 10010, USA

## Abstract

**Objectives:**

Injection drug users (IDUs) are at high risk for HIV, hepatitis, overdose and other harms. Greater drug treatment availability has been shown to reduce these harms among IDUs. Yet, little is known about changes in drug treatment availability for IDUs in the U.S. This paper investigates change in drug treatment coverage for IDUs in 90 metropolitan statistical areas (MSAs) during 1993-2002.

**Methods:**

We define *treatment coverage *as the percent of IDUs who are in treatment. The number of IDUs in drug treatment is calculated from treatment entry data and treatment census data acquired from the Substance Abuse and Mental Health Service Administration, divided by our estimated number of IDUs in each MSA.

**Results:**

Treatment coverage was low in 1993 (mean 6.7%; median 6.0%) and only increased to a mean of 8.3% and median of 8.0% coverage in 2002.

**Conclusions:**

Although some MSAs experienced increases in treatment coverage over time, overall levels of coverage were low. The persistence of low drug treatment coverage for IDUs represents a failure by the U.S. health care system to prevent avoidable harms and unnecessary deaths in this population. Policy makers should expand drug treatment for IDUs to reduce blood-borne infections and community harms associated with untreated injection drug use.

## Introduction

The HIV/AIDS epidemic among injection drug users (IDUs) is a critically important public health issue in the United States (U.S.). Currently, IDUs are the third highest risk group for HIV infection [1]. The shared use of injection equipment can also transmit hepatitis B and C [2-6]. The IDU population further experiences high rates of fatal drug overdose, endocarditis, cellulitis, and abscesses [7-10]. Injection drug users experience poor health outcomes due to delayed access to effective treatment, lower adherence to care and treatment regimens, continuation of illicit drug use, depression and negative life events [11-15].

Additionally, IDUs who do not enter treatment are six times more likely to become infected with HIV than are IDUs who enter and remain in treatment [9]. Milloy and colleagues [15] reported that being unable to access treatment was independently associated with recent incarceration, daily use of heroin and borrowing used syringes. Wood and colleagues [16] concluded that IDUs' inability to access treatment was independently associated with syringe borrowing among HIV-negative IDUs at risk for HIV infection.

Thus, there is considerable evidence indicating that drug treatment for IDUs is effective in reducing these harms [14,17-25] and improving users' health outcomes [13,14,19,26-29]. Research on the effectiveness of drug treatment further indicates that increased length of time in treatment is associated with lower rates of needle sharing and HIV seropositivity [17,21,18]. Furthermore, many programs provide access to AIDS education and prevention programs, and HIV testing, and provide contacts with health care systems to those clients already infected with HIV [13,30]. The inclusion of AIDS education programs within treatment protocols is associated with increased knowledge of AIDS risks, and decreased drug use and sexual behaviors that put IDUs at risk for HIV infection and other STIs [21,25,26,29]. Treatment therefore has the potential to address a broad social and public health agenda valued in communities affected by IDU.

Research has further demonstrated that drug treatment, such as methadone maintenance treatment, is cost-effective, and is a key component for supporting harm reduction efforts among IDUs. Pollack [31] for example, explores the cost-effectiveness of methadone maintenance as an HIV prevention strategy. Epidemiological models suggest that methadone treatment is cost-effective for reducing HIV incidence at an average cost of between $100,000 and $300,000 per infection averted. This research also concludes that methadone maintenance is most cost-effective in reducing HIV prevalence when treatment reaches a large proportion of IDUs within a given community [31]. Lastly, according to a 1998 study by Harwood [32], the cost benefits of methadone maintenance treatment show that costs to society from criminal activities related to opioid use can run as high as four times more than the costs for methadone treatment.

Though a number of studies have demonstrated that drug treatment program are effective in reducing drug use and other harm among IDUs [21,33-38], relatively little research has been done on the extent to which IDUs are actually reached by drug treatment programs (i.e. drug treatment coverage). While there is no level of coverage that universally has been agreed upon in preventing HIV among IDUs [22], current levels of HIV services for IDUs vary substantially and are inadequate in preventing HIV transmission [23,24]. And, although 100% drug treatment coverage for IDUs should be the ultimate goal, it may be both unrealistic and impossible to attain, as not all IDUs want to be in treatment, and those with opioid dependence often have a high rate of relapse, cycling in and out of treatment programs.

For the purposes of this study, *coverage *is defined as the percentage of IDUs who are in drug treatment in a given time period. Treatments included in our coverage estimates are residential or ambulatory inpatient/outpatient care, detoxification services and methadone maintenance therapy at publicly- and privately-funded substance abuse agencies receiving public funds. These are facilities licensed, certified, or otherwise approved by State substance abuse agencies to provide substance abuse treatment.

In this study we extend the scope of an earlier cross-sectional study on treatment coverage [39] to include longitudinal data, and we describe change in drug treatment coverage for IDUs during 1993-2002 in 90 metropolitan statistical areas (MSAs). [NOTE: This study considers data collected prior to the availability of buprenorphine and naltrexone in the U.S.] Research on treatment coverage can provide insight to public policy planners, treatment providers and harm reduction activists regarding geographic areas in need of drug treatment expansion.

## Methods and data

### Overview

We calculated treatment coverage rates using two data series from the Substance Abuse and Mental Health Service Administration (SAMHSA), and estimates of IDUs from previous research [40]. Below we describe each data series:

1) Proportion of treatment entrants who indicated that they injected substances intravenously in each MSA and year as reported by the Treatment Episode Data Set (TEDS) [41];

2) Total number of drug users in drug treatment as of October 1 of each year reported by the Uniform Facility Data Set (UFDS) for 1993, 1995, 1996-1998 and the National Survey of Substance Abuse Treatment Services (N-SSATS) for 2000 and 2002 [42];

3) Total number of IDUs in each MSA and year as reported by Brady and colleagues [40].

We determine changes in coverage within each MSA where treatment coverage values were regressed on calendar year.

#### Unit of Analysis and Sample

We studied 90 MSAs as defined by the US Census as of 1993 [43]. MSAs are defined as a set of contiguous counties that contain a central city of 50,000 people or more and form a socioeconomic unit determined according to commuting patterns and social and economic integration within the constituent counties.

#### Data

We define treatment coverage as the ratio of IDUs in treatment to IDUs in the MSA. Treatment coverage for IDUs is estimated using TEDS and UFDS/N-SSATS. Each of our data sets differs in counts of substance abuse treatment clients. TEDS counts each admission in a given year. Therefore, an individual admitted to treatment twice in a calendar year is counted as two admissions, which inflates annual treatment entries. In contrast, UFDS/N-SSATS is a one day census of treatment, but does not report on the route of administration. By multiplying the percent of entrants who are IDUs (from TEDS) by the UFDS/N-SSATS census total we can estimate the number of IDUs in treatment. The proportion of entrants who are IDUs is only biased to the extent that double-counting of entrants varies systematically by route of administration.

The TEDS data series identifies the number and attributes of clients who enter substance abuse treatment programs. From TEDS, we calculated the proportion of treatment entrants who reported they injected drugs as a mode of administration. Our second SAMHSA data source comes from the annual census of drug treatment facilities originally referred to as the Uniform Facility Data Set (UFDS) - but since renamed the National Survey of Substance Abuse Treatment Services (N-SSATS). UFDS/N-SSATS data measure client characteristics and use of privately- and publicly-funded substance abuse treatment programs in the U.S. on October 1 for each year. However, UFDS/N-SSATS data were unavailable for 1992, 1994, 1999, and 2001. As a result of this limited availability, our drug treatment estimates were only created for years where data were available. Thus, our final coverage estimates only include data for 1993, 1995, 1996-1998, 2000, and 2002.

#### Calculating Number of IDUs

Because estimation of the total numbers of injectors is discussed in detail elsewhere [40], it is described only briefly here. Brady and colleagues first estimated the number of IDUs in the US each year from 1992-2002 and then apportioned these estimates to MSAs using multiplier methods. Four different types of data indicating drug injection were used to allocate national annual totals to MSAs, creating four distinct series of estimates of the number of injectors in each MSA. These estimates rely on using (1) HIV counseling and testing data from the Centers for Disease Control (CDC); (2) SAMSHA's UFDS and TEDS data; (3) CDC's diagnoses of IDUs with HIV/AIDS; and (4) an estimate derived from published estimates of the number of injectors living in each MSA in 1992 [44] and in 1998 [45]. Each series was smoothed over time using loess regression and the mean value of the four component estimates was taken as the best estimate of IDUs for that MSA and year.

Brady results indicated that nationwide IDU prevalence was relatively stable from 1992 to 2002; with a decreasing average trend across the 96 MSAs, until 2000, after which there was a slight increase; and individual MSAs deviate considerably from the average trend of the 96 MSAs. IDU prevalence varied from 30 to 348 across MSAs (mean 126.9, standard deviation 65.3, median 106.6, interquartile range 78-162) in 1992 and from 37 to 336 across MSAs (mean 110.6, standard deviation 57.7, median 96.1, interquartile range 67-134) in 2002 [40].

In order to avoid circularity, the estimated numbers of IDUs in the population used in this study modify the Brady estimates so that they do not rely on data on the numbers of IDUs in drug treatment from SAMSHA.

#### Calculating Drug Treatment Coverage Rates

Treatment coverage calculation:

$$A_{jt}=\left(\frac{B_{jt}}{C_{jt}}\right)*\left(\frac{D_{jt}}{E_{jt}}\right)*100$$

*A_jt _*= treatment coverage rate for an MSA *j *in year *t*

*B_jt _*= number of IDUs entering drug treatment as reported by TEDS for an MSA *j *in year *t*

*C_jt _*= number of IDUs and NIDUs entering drug treatment as reported by TEDS for an MSA *j *in year *t*

*D_jt _*= number of drug users entering drug treatment reported by UFDS/N-SSATS for an MSA *j *in year *t*

*E_jt _*= estimated number of IDUs for an MSA *j *in year *t*

#### Calculating Change in Drug Treatment Coverage Rates

In order to use all of the available data to estimate change and test change for statistical significance within each MSA, MSA-specific treatment coverage values were regressed on calendar year. For each of these linear regression models, the coefficient for year and its standard error was multiplied by nine to represent the total model-predicted linear change in treatment coverage from 1993 to 2002. The regression coefficient for the effect of year within each MSA was tested for significance using a two-tailed *p *< 0.05 criterion. Since our aim here was to estimate change in each MSA separately, we did not adjust significance levels for multiple testing.

## Results

### Descriptive statistics

Additional file 1: Appendix 1 presents data on treatment coverage for each MSA in 1993 and 2002 and on change in coverage over time. Coverage overall changed little from 1993 to 2002. Mean coverage was only 8.3% (standard deviation 4.28%; interquartile range 5.0%-11.0%) in 2002 (Table 1), a slight increase from 6.7% in 1993 (standard deviation 3.7%; interquartile range 4.0%-9.0%). Median treatment coverage increased only from 6.0% in 1993 to 8.0% in 2002.

**Table 1:** Estimated drug treatment coverage rates (percent in treatment) among injection drug users in 90 large US MSAs, 1993-2002

	Mean	Standard Deviation	Median	Interquartile Range	Minimum	Maximum
Treatment Coverage						

1993	6.72	3.67	6.00	4.00, 9.00	1.00	16.00

1995	6.69	4.18	5.67	3.51, 9.51	0.86	20.68

1996	6.65	4.04	5.03	3.42, 9.62	0.88	18.14

1997	6.35	4.10	5.41	3.32, 9.04	0.09	20.73

1998	7.73	5.28	6.54	3.64, 11.04	0.00	26.20

2000	7.34	4.49	6.23	3.81, 9.84	0.43	23.78

2002	8.27	4.28	8.00	5.00, 11.00	1.00	21.00

Change†††	1.58	3.43	1.37	-0.57, 3.08	-9.44	10.65

NOTE: All values are percentages

††† from 1993-2002 as predicted by linear regression model

### Linear Regression Estimates of Change Over time

The regression estimates of change also depict slow growth in overall coverage during the study period (mean = 1.58%; median = 1.37%; standard deviation 3.43%; interquartile range -0.57% to 3.08%).

Fifteen MSAs experienced statistically significant increases in treatment coverage; of those, seven experienced increases of less than 5% (Atlanta, GA; Cleveland, OH; Dayton-Springfield, OH; Grand Rapids-Muskegon, MI; Miami, FL; Rochester, NY; West Palm Beach, FL). Seven MSAs experienced significant increases of between 5% and 10% (Detroit, MI; El Paso, TX; Monmouth-Ocean, NJ; Nassau-Suffolk, NY; New York, NY; Newark, NJ; Wilmington-Newark, DE-MD), and one MSA increased greater than 10% (Albany-Schenectady, NY). Three MSAs exhibited a statistically significant decrease in coverage ranging between -3.9 to -7.1 (Tacoma, WA; Fresno, CA; Honolulu, HI). Fully 80% of MSAs (N = 72) in the study experienced no statistically significant change in treatment coverage over time.

## Discussion

The overall level of treatment coverage for IDUs in our 90 MSAs began low and remained low through the end of the study period. Our results indicate there was a slight increase in average drug treatment coverage from 6.7% to 8.3% across 90 MSAs from 1993 to 2002. Fifteen MSAs experienced statistically significant increases in treatment coverage. Of those, only eight MSAs experienced increases of 5% or more (and all increases were <11%). Three MSAs exhibited a statistically significant decline in coverage. During this period, estimated treatment coverage in 72 MSAs did not change significantly over time.

Some of the statistically significant changes (e.g., Cleveland, OH; Dayton-Springfield, OH; Miami, FL) were small in size. The fact that Cleveland and other MSAs had significant increases (< 5%) while Jersey City and Providence-Fall River (> 5%) did not is due to differences in the standard variation. The authors note that several of the statistically significant increases are quite small in magnitude, but are otherwise statistically reliable with regard to treatment coverage change over time.

Although outpatient methadone maintenance has been shown to be an effective treatment modality for opioid dependent patients by 1997, only 7% of all treatment facilities offered outpatient methadone treatment, representing approximately 14-15% of all clients in the treatment system. Of these, 30% of outpatient methadone maintenance facilities are private for-profit, 53% are private non-profit, and 18% are publicly owned [46].These figures remain unchanged in 2008, with only 8% of all substance abuse treatment facilities offering some type of opioid replacement therapy, either methadone or buprenorphine [47].

The need for increased investment in drug abuse treatment programs was underscored in the 2008 National Survey on Drug Use and Health, where 3.0% of the total population (i.e., 7.6 million persons aged 12 or older) were estimated to be in need of treatment for an illicit drug use problem. Of those individuals, 1.2 million or 0.5% of the total population received treatment -- yielding a 16% coverage rate for drug users overall. This contrasts with only 8% for IDUs. Thus, an estimated 2.5% of the population (6.4 million) in need of treatment for an illicit drug use problem did not receive treatment, a figure that remains unchanged since 2002 [48]. A greater investment in treatment will likely be needed to have a substantial impact on injection drug use and associated harms. Investment in drug treatment expansion should be viewed as sound public health policy.

## Limitations

Certain limitations must be taken into account when interpreting data from TEDS and UFDS/N-SSATS. During our study period, SAMSHA eliminated questions from UFDS about the number of IDUs in treatment programs after 1998. We therefore multiplied the proportion of drug users entering treatment who inject drugs (from TEDS) in each MSA and year by the total number of drug users in treatment as reported by both UFDS/N-SSATS. Second, these data sets differ in what they count: TEDS counts each admission in a given year, so an individual who enters drug treatment twice or more in a year is counted as two or more independent cases. In contrast, UFDS/N-SSATS client count numbers are a point-prevalence survey--those in treatment on one specific day. It gives a snap-shot of what the substance abuse treatment system looks like on an average day. Consequently, if IDUs differ from non-IDUs in the ratio of admissions to those remaining in treatment, our estimates will be biased.

Additionally, change in our estimated numbers of IDUs in treatment in an MSA might in part result from change in which treatment facilities in an MSA respond to SAMSHA surveys. The survey response rate increased from 87% in 1995 to 96% in 2002, producing an 11% increase in reported U.S. client totals from 1993-2002. SAMSHA attempts to obtain responses from all known treatment facilities, but the survey is voluntary and no adjustments for facility non-response are made. Thus, the estimated increases in treatment coverage may partially reflect changes in survey methodology.

Finally, IDUs estimates beyond 2002 are currently being updated and were not readily available for our coverage estimates. Thus, our data does not extend beyond 2002. Nevertheless, there is little evidence to suggest that the number of IDUs in MSAs and the number of IDUs in treatment among the MSAs has significantly changed since then.

## Conclusions

The evidence in this paper clearly indicates the need to expand drug treatment for IDUs. Coverage may be even lower in non-metropolitan suburbs or rural areas, since they tend to have fewer available healthcare services.

Injection drug use poses serious public health challenges with respect to public policy. Low treatment coverage for IDUs may produce a high cost to society in terms of the spread of HIV, hepatitis B and C and other infectious diseases among injectors, their partners, and the broader community. Drug treatment such as methadone maintenance therapy can address a broad social and public health agenda valued in communities affected by IDU. The persistent low drug treatment coverage for IDUs represents a failure of the U.S. healthcare system to prevent avoidable harms and unnecessary deaths.

More research is needed to address what policy and structural changes affect variations and changes in treatment coverage - and, in particular, what combinations of factors lead to increases in treatment coverage. Such research can help public policy planners, treatment providers and harm reduction activists understand the barriers to expanded drug treatment for IDUs.

Coverage rates in U.S. MSAs during the study period are far below international standards. Some European Union (EU) countries maintain coverage levels of 60% or higher [49]. The World Health Organization [22] has set targets for universal access to HIV prevention, treatment and care for IDUs. Targets for treatment coverage for IDUs accessing opioid substitution therapy define " < 20%" as "low" and " > 40%" as "high [22]." Switzerland, where opioid substitution therapy is freely available, has attained treatment coverage levels of up to 70% over time [50]. Treatment coverage rates in the EU and other countries (e.g. Australia, 50%) are higher in comparison with U.S. Clearly, more funding for drug treatment services for IDUs is needed from U.S. government sources to increase the overall level of treatment coverage to a level comparable to what the EU and other countries attain. Reducing the harm associated with injection drug use and making drug treatment accessible and a high public health priority is sound public health and government policy.

## Competing interests

The authors declare that they have no competing interests.

## Authors' contributions

BT was responsible for data acquisition, analysis, and interpretation; and the writing of the article. CMC contributed to the analysis and interpretation of the data; and the writing of the article. SRF contributed to the conception and design of the analysis and the writing. ERP contributed to the writing of the article. SC contributed to data acquisition. BT, CMC, SRF, ERP, and SC read and approved the final manuscript.

## Supplementary Material

Additional file 1 **Appendix 1**. Distribution and Change in Estimated Drug Treatment Coverage Rates among Injection Drug Users (IDUs) 1993-2002. Click here for file
