# Supplementary material for: Persistence of low drug treatment coverage for injection drug users in large US metropolitan areas
Source: Subst Abuse Treat Prev Policy. 2010 Sep 21;5:23. doi: 10.1186/1747-597X-5-23 (PMC2954979; doi:10.1186/1747-597X-5-23)
Supplement: Additional file 1 — Appendix 1. Distribution and Change in Estimated Drug Treatment Coverage Rates among Injection Drug Users (IDUs) 1993-2002. [file 1747-597X-5-23-S1.DOC]

|  | **Number of IDUs** | | | **Number of IDUs in Treatment** | | | **Treatment Coverage**  **(Percent of IDUs in Treatment)** | | |
| --- | --- | --- | --- | --- | --- | --- | --- | --- | --- |
| **MSA** | **1993** | **2002** | **Change**  **1993-2002** | **1993** | **2002** | **Change**  **1993-2003** | **1993 %** | **2002 %** | **Change**  **1993-2002** |
| Akron, OH | 2400 | 3122 | 722 | 123 | 215 | 93 | 5.1 | 6.9 | 1.8 |
| Albany-Schenectady, NY | 3395 | 3683 | 288 | 205 | 606 | 401 | 6.1 | 16.5 | **†** 10.4 |
| Albuquerque, NM | 9207 | 9066 | -141 | 721 | 868 | 146 | 7.8 | 9.6 | 1.7 |
| Allentown-Bethlehem, PA | 5570 | 6261 | 691 | 254 | 158 | -96 | 4.6 | 2.5 | -2.0 |
| Ann Arbor, MI | 1297 | 1585 | 288 | 110 | 154 | 44 | 8.5 | 9.7 | 1.2 |
| Atlanta, GA | 19845 | 14602 | -5243 | 164 | 599 | 435 | 0.8 | 4.1 | **†** 3.3 |
| Austin-San Marcos, TX | 14067 | 9655 | -4412 | 707 | 678 | -29 | 5.0 | 7.0 | 2.0 |
| Bakersfield, CA | 10779 | 10800 | 21 | 557 | 860 | 303 | 5.2 | 8.0 | 2.8 |
| Baltimore, MD | 33040 | 58720 | 25680 | 3659 | 5566 | 1907 | 11.1 | 9.5 | -1.6 |
| Bergen-Passaic, NJ | 8547 | 6868 | -1678 | 806 | 858 | 52 | 9.4 | 12.5 | 3.1 |
| Birmingham, AL | 2811 | 3058 | 247 | 118 | 148 | 30 | 4.2 | 4.8 | 0.6 |
| Boston, MA-NH | 39743 | 67044 | 27301 | 6534 | 12851 | 6317 | 16.4 | 19.2 | 2.7 |
| Buffalo-Niagara Falls, NY | 6130 | 6197 | 67 | 762 | 912 | 149 | 12.4 | 14.7 | 2.3 |
| Charleston-North Charleston, SC | 2047 | 1928 | -119 | 85 | 76 | -9 | 4.2 | 4.0 | -0.2 |
| Charlotte-Gastonia, NC-SC | 6099 | 6035 | -64 | 173 | 302 | 129 | 2.8 | 5.0 | 2.2 |
| Chicago, IL | 31793 | 32206 | 413 | 1386 | 1301 | -85 | 4.4 | 4.0 | -0.3 |
| Cincinnati, OH-KY-IN | 6871 | 7213 | 341 | 276 | 369 | 94 | 4.0 | 5.1 | 1.1 |
| Cleveland, OH | 10370 | 10947 | 577 | 520 | 769 | 249 | 5.0 | 7.0 | **†** 2.0 |
| Columbus, OH | 7963 | 10111 | 2148 | 441 | 672 | 230 | 5.5 | 6.6 | 1.1 |
| Dallas, TX | 21277 | 31931 | 10654 | 619 | 1157 | 538 | 2.9 | 3.6 | 0.7 |
| Dayton-Springfield, OH | 3564 | 4267 | 703 | 194 | 322 | 127 | 5.5 | 7.5 | **†** 2.1 |
| Denver, CO | 19328 | 20689 | 1360 | 1707 | 2665 | 958 | 8.8 | 12.9 | 4.0 |
| Detroit, MI | 23999 | 27166 | 3167 | 2306 | 4030 | 1724 | 9.6 | 14.8 | **†** 5.2 |
| El Paso, TX | 11422 | 6616 | -4806 | 473 | 777 | 305 | 4.1 | 11.7 | **†** 7.6 |
| Fort Lauderdale, FL | 7349 | 7375 | 26 | 176 | 248 | 72 | 2.4 | 3.4 | 1.0 |
| Fort Worth-Arlington, TX | 19505 | 20192 | 687 | 555 | 1067 | 512 | 2.8 | 5.3 | 2.4 |
| Fresno, CA | 16986 | 18258 | 1272 | 2346 | 1269 | -1077 | 13.8 | 7.0 | **††** -6.9 |
| Grand Rapids-Muskegon, MI | 2665 | 3107 | 442 | 137 | 305 | 169 | 5.1 | 9.8 | **†** 4.7 |
| Greenville-Spartanburg, SC | 2384 | 2789 | 405 | 77 | 169 | 93 | 3.2 | 6.1 | 2.9 |
| Harrisburg-Lebanon, PA | 3238 | 4603 | 1365 | 172 | 280 | 108 | 5.3 | 6.1 | 0.8 |
| Honolulu, HI | 4732 | 5145 | 413 | 259 | 93 | -166 | 5.5 | 1.8 | **††** -3.7 |
| Houston, TX | 43560 | 34117 | -9443 | 686 | 743 | 57 | 1.6 | 2.2 | 0.6 |
| Indianapolis, IN | 8980 | 8712 | -269 | 721 | 712 | -8 | 8.0 | 8.2 | 0.2 |
| Jacksonville, FL | 7603 | 8278 | 675 | 129 | 241 | 112 | 1.7 | 2.9 | 1.2 |
| Jersey City, NJ | 7460 | 5899 | -1561 | 365 | 687 | 322 | 4.9 | 11.7 | **†††** 6.8 |
| Kansas City, MO-KS | 7541 | 5879 | -1662 | 422 | 408 | -14 | 5.6 | 6.9 | 1.3 |
| Knoxville, TN | 3626 | 3800 | 174 | 153 | 275 | 122 | 4.2 | 7.2 | 3.0 |
| Las Vegas, NV-AZ | 11718 | 13708 | 1989 | 390 | 754 | 364 | 3.3 | 5.5 | 2.2 |
| Little Rock, AR | 5243 | 4893 | -350 | 283 | 253 | -30 | 5.4 | 5.2 | -0.2 |
| Los Angeles-Long Beach, CA | 103900 | 98616 | -5285 | 10879 | 10070 | -809 | 10.5 | 10.2 | -0.3 |
| Louisville, KY-IN | 6817 | 10136 | 3319 | 189 | 221 | 32 | 2.8 | 2.2 | -0.6 |
| Memphis, TN-AR-MS | 5082 | 5881 | 799 | 66 | 57 | -10 | 1.3 | 1.0 | -0.3 |
| Miami, FL | 14553 | 9280 | -5273 | 396 | 444 | 48 | 2.7 | 4.8 | **†** 2.1 |
| Middlesex-Somerset, NJ | 5612 | 4984 | -628 | 584 | 598 | 14 | 10.4 | 12.0 | 1.6 |
| Milwaukee-Waukesha, WI | 4871 | 5696 | 825 | 289 | 280 | -9 | 5.9 | 4.9 | -1.0 |
| Minneapolis-St. Paul, MN-WI | 8265 | 10297 | 2032 | 363 | 319 | -44 | 4.4 | 3.1 | -1.3 |
| Monmouth-Ocean, NJ | 5762 | 6087 | 325 | 400 | 970 | 569 | 7.0 | 15.9 | **†** 9.0 |
| Nashville, TN | 5990 | 8728 | 2738 | 154 | 93 | -61 | 2.6 | 1.1 | -1.5 |
| Nassau-Suffolk, NY | 9861 | 12177 | 2316 | 905 | 2299 | 1395 | 9.2 | 18.9 | **†** 9.7 |
| New Orleans, LA | 13446 | 14415 | 969 | 414 | 467 | 53 | 3.1 | 3.2 | 0.2 |
| New York, NY | 111618 | 91327 | -20292 | 10020 | 13186 | 3166 | 9.0 | 14.4 | **†** 5.5 |
| Newark, NJ | 19705 | 16153 | -3552 | 1268 | 2027 | 759 | 6.4 | 12.5 | **†** 6.1 |
| Norfolk-Virginia Beach, VA-NC | 8098 | 10259 | 2161 | 563 | 575 | 12 | 6.9 | 5.6 | -1.3 |
| Oakland, CA | 26868 | 22259 | -4609 | 2925 | 2430 | -494 | 10.9 | 10.9 | 0.0 |
| Oklahoma City, OK | 7460 | 6236 | -1223 | 927 | 606 | -321 | 12.4 | 9.7 | -2.7 |
| Omaha, NE-IA | 2608 | 3342 | 734 | 232 | 327 | 95 | 8.9 | 9.8 | 0.9 |
| Orange County, CA | 24973 | 18915 | -6058 | 3100 | 2705 | -395 | 12.4 | 14.3 | 1.9 |
| Orlando, FL | 6260 | 10890 | 4630 | 294 | 706 | 412 | 4.7 | 6.5 | 1.8 |
| Pittsburgh, PA | 10593 | 14268 | 3675 | 627 | 1361 | 735 | 5.9 | 9.5 | 3.6 |
| Portland-Vancouver, OR-WA | 23236 | 26735 | 3498 | 2483 | 3357 | 874 | 10.7 | 12.6 | 1.9 |
| Providence-Fall River, RI-MA | 5901 | 7888 | 1987 | 826 | 1648 | 822 | 14 | 20.9 | **†††** 6.9 |
| Raleigh-Durham, NC | 5528 | 4630 | -898 | 127 | 180 | 52 | 2.3 | 3.9 | 1.6 |
| Richmond-Petersburg, VA | 6729 | 6753 | 24 | 371 | 511 | 139 | 5.5 | 7.6 | 2.0 |
| Riverside-San Bernardino, CA | 32172 | 20525 | -11647 | 3688 | 2213 | -1474 | 11.5 | 10.8 | -0.7 |
| Rochester, NY | 5000 | 6029 | 1028 | 265 | 616 | 351 | 5.3 | 10.2 | **†** 4.9 |
| Sacramento, CA | 18887 | 14871 | -4016 | 2062 | 1229 | -833 | 10.9 | 8.3 | -2.7 |
| Salt Lake City-Ogden, UT | 7361 | 10770 | 3409 | 696 | 1218 | 522 | 9.5 | 11.3 | 1.8 |
| San Antonio, TX | 19746 | 14683 | -5064 | 1492 | 996 | -496 | 7.6 | 6.8 | -0.8 |
| San Diego, CA | 28206 | 25946 | -2260 | 3379 | 2919 | -460 | 12.0 | 11.3 | -0.7 |
| San Francisco, CA | 32032 | 28462 | -3571 | 3532 | 2884 | -648 | 11.0 | 10.1 | -0.9 |
| San Jose, CA | 13831 | 6493 | -7338 | 963 | 547 | -416 | 7.0 | 8.4 | 1.5 |
| Sarasota-Bradenton, FL | 2569 | 4601 | 2032 | 150 | 255 | 105 | 5.9 | 5.5 | -0.3 |
| Scranton-Wilkes-Barre, PA | 1729 | 2199 | 470 | 198 | 312 | 114 | 11.5 | 14.2 | 2.7 |
| Seattle-Bellevue, WA | 29440 | 28505 | -935 | 4134 | 3412 | -721 | 14.0 | 12.0 | -2.1 |
| Springfield, MA | 6071 | 9193 | 3121 | 687 | 1233 | 547 | 11.3 | 13.4 | 2.1 |
| St. Louis, MO-IL | 12211 | 10942 | -1269 | 437 | 445 | 7 | 3.6 | 4.1 | 0.5 |
| Stockton-Lodi, CA | 7175 | 10976 | 3801 | 459 | 964 | 505 | 6.4 | 8.8 | 2.4 |
| Syracuse, NY | 2594 | 2100 | -494 | 110 | 168 | 58 | 4.2 | 8.0 | 3.8 |
| Tacoma, WA | 7813 | 7988 | 176 | 1264 | 726 | -538 | 16.2 | 9.1 | **††** -7.1 |
| Tampa-St. Petersburg, FL | 12581 | 16455 | 3874 | 321 | 911 | 591 | 2.6 | 5.5 | 3.0 |
| Toledo, OH | 2251 | 3172 | 921 | 132 | 134 | 2 | 5.8 | 4.2 | -1.6 |
| Tulsa, OK | 4071 | 4840 | 769 | 190 | 265 | 75 | 4.7 | 5.5 | 0.8 |
| Ventura, CA | 5793 | 5873 | 79 | 736 | 799 | 62 | 12.7 | 13.6 | 0.9 |
| Washington, DC-MD-VA-WV | 23587 | 28237 | 4650 | 1051 | 2199 | 1148 | 4.5 | 7.8 | 3.3 |
| West Palm Beach, FL | 7219 | 7344 | 125 | 137 | 456 | 320 | 1.9 | 6.2 | **†** 4.3 |
| Wichita, KS | 1863 | 2678 | 815 | 223 | 284 | 61 | 12.0 | 10.6 | -1.4 |
| Wilmington-Newark, DE-MD | 4225 | 6943 | 2718 | 100 | 721 | 622 | 2.4 | 10.4 | **†** 8.0 |
| Youngstown-Warren, OH | 1227 | 2386 | 1159 | 82 | 105 | 23 | 6.7 | 4.4 | -2.3 |
